# Feasibility of aortic valve assessment with low dose prospectively triggered adaptive systolic (PTAS) cardiac computed tomography angiography

**DOI:** 10.1186/1756-0500-6-158

**Published:** 2013-04-20

**Authors:** Ashley M Lee, Jonathan Beaudoin, Wai-Ee Thai, Bryan Wai, Gladwin C Hui, Manavjot S Sidhu, Leif-Christopher Engel, Suhny Abbara, Udo Hoffmann, Brian B Ghoshhajra

**Affiliations:** 1 Department of Radiology and Division of Cardiology, Cardiac MR PET CT Program, Massachusetts General Hospital and Harvard Medical School, 165 Cambridge St, Suite 400, Boston, MA, 02114, USA

**Keywords:** Computed tomography angiography, Aortic stenosis, Low dose

## Abstract

**Background:**

Cardiac computed tomography angiography (CTA) is feasible for aortic valve evaluation, but retrospective gated protocols required high radiation doses for aortic valve assessment. A prospectively triggered adaptive systolic (PTAS) cardiac CT protocol was recently described in arrhythmia using second-generation dual-source CT. In this study, we sought to evaluate the feasibility of PTAS CTA to assess the aortic valve at a low radiation dose.

**Findings:**

A retrospective cohort of 29 consecutive patients whom underwent PTAS protocols for clinical indications other than aortic valve assessment and whom also received echocardiography within 2 months of CT, was identified. Images were reviewed for aortic valve morphology (tricuspid/bicuspid/prosthetic) and stenosis (AS) by experienced blinded readers. Accuracy versus echocardiography and radiation doses were assessed.

All PTAS coronary CTAs were clinically diagnostic with 0 un-evaluable coronary segments. The accuracy of PTAS for aortic valve morphology was 92.6%, and for exclusion of severe AS was 93.1%. Two exams were un-evaluable for the aortic valve due to inadequate number of phases archived for interpretation. Total radiation dose was a median of 2.8 mSv (interquartile range 1.4–4.4 mSv).

**Conclusions:**

PTAS CTA protocols using second-generation dual-source CT for aortic valve evaluation are feasible at low doses. This protocol should be investigated further in larger cohorts.

## Findings

Prospectively ECG-triggered cardiac CT angiography targeted to end-systole for the evaluation of aortic pathology was feasible at a low radiation dose (2.8 mSv) with 128 dual-source CT.

### Introduction

The feasibility of cardiac computed tomography angiography (CTA) for aortic valvular evaluation has been established for aortic stenosis (AS) evaluation (via direct planimetry) in small referral cohorts [1-9], and has performed well versus transthoracic echocardiography. All initially published studies were performed using retrospective ECG gating to allow systolic phase evaluation of aortic valve opening [10,11]. Retrospective ECG gating is widely available but involves significant radiation dose expense versus prospective triggering and is now used infrequently [12,13].

Various refinements to prospective triggering algorithms such as arrhythmia rejection are available [13-15]. Recently, the use of a systolic target for prospective triggering was described as a means to mitigate variable and elevated heart rates [16-18]; this method was applied using late-generation dual-source CT scanners, which allowed a “systolic absolute delay” to specify an acquisition in systole after the R-peak, and a fixed range of data acquisition to enable various reconstruction intervals to achieve motion-free images. This method limits imaging to mid and late systole, phases shown to be ideal for aortic valve area opening measurements [19].

Because this protocol may allow aortic valve analysis (valve morphology and AS), we evaluated its feasibility versus echocardiography. We hypothesized that prospectively triggered adaptive systolic images (PTAS) would allow adequate aortic valve evaluation at a significantly decreased radiation dose versus retrospectively ECG-gated images (6.7 to 20 mSv in one meta-analysis) [11].

### Technical methods

Our institutional IRB (The Partners Human Research Committees) granted a waiver for this retrospective research. HIPAA compliance was maintained throughout the study. No outside funding was received, and the authors maintained full control over the data.

#### Patient population

This retrospective study included 29 patients who underwent clinically indicated CTA with PTAS from December 2011 to August 2012. All patients underwent echocardiography within two months of CTA.

#### Data acquisition

**Figure 1:**
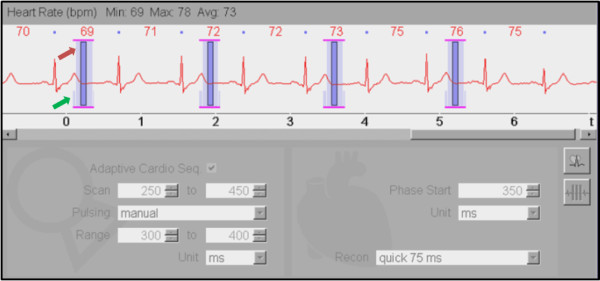
**ECG strip demonstrating timing of image acquisition.** PTAS CTA is shown with radiation exposure peak at 100% of the reference tube current (red arrow, “Range” settings) at 300–400 msec and a “plateau” (green arrow, “Scan” settings) with 20% of the reference tube current to capture additional phases at mid and late systole/early diastole.

All exams were performed on a second-generation dual source 128-slice CT scanner (SOMATOM Definition Flash, Siemens Medical Systems with software update VA40, Forchheim, Germany). A gantry rotation time of 280 milliseconds (msec) yielded a temporal resolution of 75 msec. Sublingual nitroglycerin (0.6 mg) was given unless contraindicated (i.e. known aortic stenosis at the time of CT scan). Beta-blocker (metoprolol IV) was administered per physician discretion when necessary. Contrast was delivered (Iopamidol 370 g/cm3, Isovue 370, Bracco Diagnostics, Princeton, NJ USA) via power injector per clinical routine. An automatic tube potential selection with tube current modulation algorithm [20,21] was used to minimize radiation dose while maintaining diagnostic image contrast-to-noise ratio. The PTAS protocol was executed with a positive absolute delay of 300–400 msec after the R peak (100% mAs exposure, Figure 1) with widened window acquisition (20% mAs, 200–300 msec and 400–450 msec). Arrhythmia rejection algorithm (Adaptive Cardio Sequence, “Adaptive Cardio Sequential Flex mode”, Siemens Medical Systems, Forchheim, Germany) was enabled in all scan acquisition. This arrhythmia rejection algorithm compensated for gradual changes in heart rate and allowed scan positions to be immediately repeated in the case of an ectopic or widely irregular beat.

#### Image reconstruction and analysis

Raw data were reconstructed at multiple phases of R-R interval (300 msec, 350 msec, 375 msec, 400 msec, and/or as clinically necessary). For each phase archived in PACS, images were reconstructed in multiplanar reformatted axes to obtain a series of short axis view through the aortic valve.

#### Radiation exposure

Volume CT dose index (CTDI_vol_) and dose length product (DLP) for the CTA scans were recorded and effective dose was calculated [12].

#### Aortic valve evaluation

Two readers each with 4 years each of advanced training (2 years of echocardiography and 2 additional years of cardiac CT fellowships) were blinded to CTA and echocardiography results. Readers reviewed images in consensus and assessed aortic valve morphology and AS. The aortic valve was assessed as 1) tricuspid, 2) bicuspid, or 3) prosthetic. Confidence in their assessment was graded on a 4-point Likert scale (1 = not at all, 2 = somewhat, 3 = confident, 4 = very confident). Aortic valve orifice area was measured for each available phase [2,11]. The maximum area was used to grade potential AS (no evidence of severe AS vs. severe AS [area less than 1 cm^2^ by planimetry]) [2,4]. If severe AS could be excluded, confidence was graded in a similar 4-point Likert scale. Clinical echocardiography reports were independently reviewed by a research fellow. In echocardiography, aortic valve area was evaluated by the continuity equation with valve area less than 1 cm^2^ considered severe stenosis. Agreement tables between PTAS CTA and echocardiography were compared.

#### Statistical analysis

Parametric variables were expressed in mean ± standard deviation. Non-parametric variables were expressed in median and interquartile range (IQR). Categorical variables were expressed as percentages. The accuracy of PTAS CTA was calculated by (number of cases with result agreement between CTA and echocardiography) / (total number of cases evaluable by echocardiography).

### Technical results

Of these 29 patients, most (19/29) were referred for native coronary assessment (Table 1). The mean heart rate was 66.4 ± 10.9 beats per minute (bpm) and average heart rate variability (maximum heart rate–minimum heart rate) was 39.9 ± 35.6 bpm. Table 1 lists detailed patient characteristic and scan parameters.

**Table 1:** Patient characteristics

**Patients characteristics and scan parameters n = 29**	
Age	64.1 ± 16.6
Male	18 (62.1%)
Mean heart rate (bpm)	66.4 ± 10.9
Heart rate variability (bpm)	39.9 ± 35.6
Rhythm during scan	
Sinus rhythm	19 (65.5%)
Atrial fibrillation	7 (24.1%)
Other (atrial flutter, etc.)	3 (10.3%)
Contrast amount (cc)	96.1 ± 13.9
Flow rate (cc/sec)	6.0 ± 2.8
Beta blocker used	17 (58.6%)
BMI	24.9 ± 4.6

All PTAS CTAs were clinically diagnostic with 0 un-evaluable coronary and bypass segments with respect to motion artifact. The average effective dose was 2.8 [1.4–4.4] mSv for all scans. Refer to Table 2 for detailed radiation parameters by tube potential and scan indication.

**Table 2:** PTAS CTA radiation exposure by tube potential and scan indication

	**Tube current (mAs)**	DLP (mGy*cm)	**CTDI**_**vol **_**(mGy)**	**Scan length (cm)**	**Effective Radiation (mSv)**
**All exams**	218.0 ± 40.2	201.0 [98.3–311.3]	12.9 [6.2–20.7]	17.2 [13.8–20.6]	**2.8 [1.4–4.4]**
**Radiation exposure by tube voltage**					
**80 kV (n = 8)**	211.4 ± 42.0	88.0 [85.0–105.5]	6.1 [4.9–6.3]	17.2 [13.8–17.2]	**1.2 [1.2–1.5]**
**100 kV (n = 10)**	202.8 ± 36.8	189.0 [140–231]	12.1 [6.8–13.2]	15.6 [13.8–20.6]	**2.6 [2.0–3.2]**
**120 kV (n = 7)**	222.1 ± 35.3	298.0 [256.5–385.5]	20.7 [18.2–21.6]	13.8 [13.8–17.3]	**4.2 [3.6–5.4]**
**140 kV (n = 4)**	262.0 ± 29.7	768.0 [642.5–872.0]	39.0 [36.3–40.6]	20.7 [17.2–22.4]	**10.8 [9.0–12.2]**
**Radiation exposure by scan indication**					
**Native coronary (n = 19)**	225.7 ± 40.1	157.0 [86.0–296.3]	9.9 [6.0–21.1]	13.8 [13.8–17.2]	**2.2 [1.2–4.1]**
**Bypass graft (n = 3)**	232.0 ± 17.1	713.0 [319.3–869.0]	34.5 [22.2–37.2]	20.7 [12.9–23.3]	**10.0 [4.5–12.2]**
**Aorta (n = 5)**	183.2 ± 35.1	231.0 [202.3–292.5]	13.2 [9.8–15.1]	20.6 [17.3–20.7]	**3.2 [2.8–4.1]**
**Pre-TAVI (n = 2)**	211.0 ± 53.7	180 [90–270]	8.7 [4.3–13.0]	20.7 [ 20.7–20.7]	**2.5 [1.3–3.8]**

**Figure 2:**
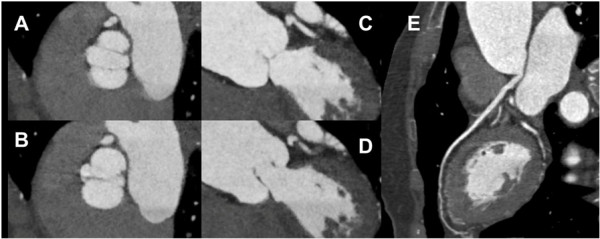
**Bicuspid aortic valve.** The cine clip (Additional file 1) shows the open valve (**A**, **C**) at 250 mm and closed valve (**B**, **D**) at 400 msec. Curved MPR of the normal LAD without stenosis (**E**).

**Figure 3:**
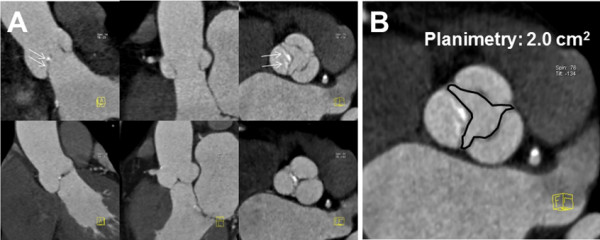
**Example of PTAS CTA in aortic valve evaluation. A**: Reconstructions at 250 msec (top row) and 400 msec (bottom row) show the aortic valve in open and closed positions. There is mild restriction of the non-coronary cusp opening (arrows). **B**: Planimetry shows mild aortic stenosis. No motion artifacts were seen in the coronary arteries (not shown).

The accuracy of PTAS CTA in assessment of aortic valve morphology was 92.6% versus echocardiography (Table 3, Figures 2 and 3, Additional file 1), with a reader confidence of 3.2 ± 1.1 (confident).

**Table 3:** Valve morphology agreement table between PTAS CTA and echocardiography

		**Echocardiography**			
		**Tricuspid**	**Bicuspid**	**Prosthetic**	**Not assessed/ not evaluated**
**PTAS CTA**	Tricuspid	21			2
	Bicuspid		1		
	Prosthetic			3	
	Cannot evaluate	2			

The accuracy of PTAS CTA to exclude severe AS was 93.1% versus echocardiography (Tables 4 and 5), with a reader confidence of 3.4 ± 1.1 (between confident and very confident).

**Table 4:** AS agreement table between PTAS CTA and echocardiography

		**Echocardiography**		
		**No severe aortic stenosis**	**Severe aortic stenosis**	**Not reported**
**PTAS CTA**	No severe aortic stenosis	23		
	Severe aortic stenosis		4	
	Un-evaluable	2		

**Table 5:** Overall performance of PTAS CTA on aortic valve evaluation

	**Accuracy**	**Confidence**
**Valve morphology**	92.6%	3.2 ± 1.1
**Severe aortic stenosis**	93.1%	3.4 ± 1.1

Reasons for low confidence were similar for aortic valve morphology and AS assessment, such as that the aortic valve remained in closed position throughout all phases (n = 2 for both), motion artifact (n = 1 for valve morphology, n = 2 for AS assessment), high image noise (n = 1 for both), and poor contrast opacification (n = 1 for both).

### Discussion

We evaluated the feasibility of a novel protocol to allow prospectively ECG-triggered cardiac CT angiography targeted to systolic phases for secondary evaluation of aortic valvular pathology. We demonstrated that when adequate phases of the R-R interval were available, the aortic valve could be evaluated with low radiation doses (2.8 [1.4–4.4] mSv). An important caveat in this analysis is that patients were referred primarily for coronary arterial or bypass graft (n = 21) and aortic root or annulus (n = 5) evaluation (with fully diagnostic results for all primary indications).

PTAS CTA was initially described as a means of limiting radiation dose in the setting of heart rate irregularity [16-18]. Previously, *Feuchtner et al* demonstrated the feasibility of assessing valvular function and morphology in patients with stable sinus rhythm. However, we note that in that study, only patients with high heart rate (>65 bpm) received systolic triggered exams with widened window acquisition (patients with low heart rate underwent diastolic triggered exams). In addition, those CTAs were performed with additional 20% mAs exposure during 10–90% of the cardiac cycle. In our present study, the widened acquisition window with 20% mAs exposure was only used from 200 to 450 msec after the R peak, or approximately 20%–50% of the cardiac cycle for patients with a heart rate of 66 bpm (the average heart rate in our cohort). Therefore, while *Feuchtner et al* showed accurate aortic assessment with 20% mAs exposure during nearly the entire cardiac cycle, we demonstrated that systolic phase image acquisition with a 250 msec acquisition window may be sufficient for aortic assessment.

The two most effective methods of cardiac CT dose reduction are the use of prospective triggering and tube potential lowering [13,14,22]. Traditionally, prospectively triggered CTAs were prone to motion or misregistration artifacts caused by irregular heart rhythms. In fact, atrial fibrillation was formerly a relative contraindication to CTA due to high radiation exposure and image quality concerns [23]. This challenge posed by elevated and irregular heart rates and rhythms has been progressively addressed by technologic developments such as arrhythmia rejection algorithms [16] and systolic triggering [17].

In addition to prospective ECG triggering, we implemented automatic kVp selection to minimize tube voltage and concomitantly adjust tube current to preserve the necessary photon flux [20,21]. Some cases involved higher kV settings due to large patients and longer scans of the entire chest since the scans were not clinically performed for aortic valve assessment.

Concurrent assessment of aortic valve by PTAS CTA in addition to coronary assessment carries several clinical advantages. If aortic valve pathology is unknown or unexpected, obvious benefits can be realized through early diagnosis. In cases of thoracic aortic evaluation, the questions of aortic valve morphology (such as bicuspid versus tricuspid) might be addressed, thereby obviating transesophageal echocardiography in some cases. In the setting of known aortic valvular disease (such as in percutaneous aortic valve implantation planning CTA), a PTAS protocol can allow concurrent valvular assessment and coronary artery stenosis exclusion. This could obviate the need for preoperative invasive coronary angiography (acknowledging that these are older, higher risk patients than traditional CTA patients), and potentially obviate the need for pre-procedural transesophageal echocardiography. While the traditional assessment of the significance of aortic stenosis is by Doppler evaluation, direct planimetry is performed by transesophageal echocardiography; this can be assessed by CTA [10].

Our retrospective cohort study carries several limitations precisely because patients were referred for other indications than primary aortic valve assessment. While images were sufficient for the clinically requested coronary or aortic vascular assessment in all cases, insufficient aortic valvular phase images were archived in 2 cases. In addition, although adequate imaging may have been initially acquired for aortic valve analysis, not all necessary phases were available for interpretation in our study (i.e. were not archived). Second, we utilized a second-generation dual-source scanner, with the most current software upgrade. Other scanners have been used for aortic valve imaging, but may not have available similar settings such as prospective triggering options with positive absolute phase start times or high temporal resolution [19]. Other equipment therefore may require different triggering settings or require longer acquisitions and larger radiation doses. Our method therefore warrants dedicated confirmatory study in a larger cohort on this specific scanner, and exploratory study using other scanner models which offer prospective ECG triggering.

### Conclusion

We demonstrated feasibility of a prospectively ECG-triggered cardiac CT angiography targeted to end-systole for the evaluation of aortic pathology at a low radiation dose (2.8 mSv) with echocardiography as the reference standard. This is significantly lower than previously reported retrospectively ECG-gated modes (6.7 to 20 mSv). We note that care must be taken to reconstruct sufficient phase reconstructions to completely evaluate the aortic valve. Our results suggest that further study in a larger cohort should be performed to confirm our favorable results and to ensure the maintained diagnostic capacity of native coronary arteries.

### Consent

Our institutional IRB (The Partners Human Research Committees) granted a waiver for this retrospective research. Patient consent was not necessary due to the retrospective nature of this study.

## Availability of supporting data

Data available upon request.

## Competing interests

Authors report no financial or non-financial competing interest.

## Authors’ contributions

All authors contributed extensively to the work presented in this paper. Specifically, the concept and technique was developed by BBG, UH, and SA. WH and BW were the readers. AML, JB, LE, GCH, and MS were responsible for literature research, statistical analysis, and manuscript drafting. All authors were responsible for manuscript supervision and revision. All authors have read and approved the final manuscript.

## Supplementary Material

Additional file 1 Cine clip showing the opening and closing of aortic valve. Click here for file
