# Supplementary figures and images for: Feasibility of aortic valve assessment with low dose prospectively triggered adaptive systolic (PTAS) cardiac computed tomography angiography
Source: BMC Res Notes. 2013 Apr 20;6:158. doi: 10.1186/1756-0500-6-158 (PMC3640955; doi:10.1186/1756-0500-6-158)

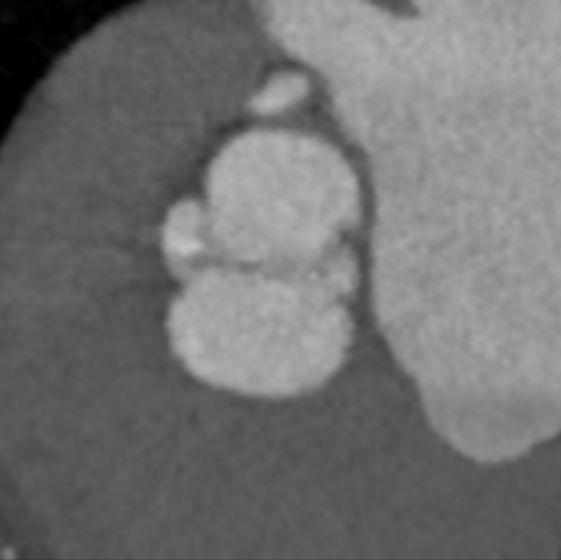

Supplement: Additional file 1 — Cine clip showing the opening and closing of aortic valve. [file 1756-0500-6-158-S1.gif]
